# Biofilm-producing and specific antibiotic resistance genes in *Pseudomonas aeruginosa* isolated from patients admitted to a tertiary care hospital, Bangladesh

**DOI:** 10.1016/j.ijregi.2024.100369

**Published:** 2024-04-25

**Authors:** Rubaiya Binte Kabir, Tasnim Ahsan, Md. Faizur Rahman, Mohammad Jobayer, SM Shamsuzzaman

**Affiliations:** 1 Department of Microbiology, Dhaka Medical College, Dhaka, Bangladesh; 2 Department of Microbiology, Ibn Sina Medical College, Dhaka, Bangladesh

**Keywords:** Antibiotic resistance, Bangladesh, Biofilm, *Pseudomonas aeruginosa*

## Abstract

**Objectives:**

Biofilms are responsible for persistent infections and antimicrobial resistance*. Pseudomonas aeruginosa* was investigated with its ability to form biofilm by detecting genes responsible for producing biofilms and biofilm-specific antimicrobial resistance. The association between antibiotic resistance and biofilm was investigated.

**Methods:**

This cross-sectional study was conducted from July 2017 to December 2018. A total of 446 samples (infected burn, surgical wounds, and endotracheal aspirate) were collected from admitted patients of Dhaka Medical College and Hospital, Bangladesh. *P. aeruginosa* was isolated and identified by biochemical tests and polymerase chain reaction. Biofilm production by tissue culture plate method followed by detection of biofilm-producing genes (*pqsA, pslA, pslD, pslH, pelA, lasR*) and biofilm-specific antibiotic resistance genes (*ndvB*, PA1874, PA1876, PA1877) by polymerase chain reaction were done. Antibiotic susceptibility test was carried out by disk diffusion method; for colistin agar dilution method of minimal inhibitory concentration was followed.

**Results:**

Among 232 (52.02%) positive strains of *P. aeruginosa*, 24 (10.30%) produced biofilms in tissue culture plate. Among biofilm-producing genes, *pqsA* was the highest (79.17%). *pslA* and *pelA* were 70.83%*, pslD* 45.83%, *pslH* and *lasR* 37.5%. Among biofilm-specific antibiotic resistance genes, 16.67% were *ndvB*, and 8.33% were PA1874 and PA1877. Biofilm-forming strains were significantly resistant to colistin.

**Conclusions:**

Detection of biofilm-forming genes may be a good tool for the evaluation of biofilm production, which will help in prompt and better management of chronic or device-associated infections.

## Highlights

• Biofilm-forming genes are useful to identify biofilm in *Pseudomonas aeruginosa*.
• Biofilm is not necessary for antibiotic resistance in *P. aeruginosa.*
• The *pqs*A gene is the most prevalent biofilm-forming gene in *P. aeruginosa.*

## Introduction

*Pseudomonas aeruginosa* (*P. aeruginosa*) has various factors that help to adhere and damage cells, and mucosal tissues of the host, elicit inflammation, and impair defense mechanisms [1]. Biofilm is one of the factors that help in the establishment of the organism on different host tissues, especially in immunocompromised patients, patients with implanted devices, and burn wounds [2,3].

Biofilm is a network among multilayered cell clusters that acts as a protective barrier against the host immune system and antibiotic therapy [4]. *P. aeruginosa* produces three extracellular polysaccharides (EPS): alginate, polysaccharide (Psl), and pellicle), that help in biofilm formation [5]. Quorum sensing (QS), a cell-to-cell signaling system, aids the production of different virulent factors causing chronic infection [6]. The pqs QS system uses *Pseudomonas* quinolone signal (PQS) as the signal molecule acting as an ideal anti-virulence drug target [7]. In the Las QS system, LasR (elastase) gene acts as a transcriptional activator that encodes virulence factors like protease, elastase, hydrogen cyanide, and phenazines [8].

Tolerance to killing by antimicrobials is the hallmark of biofilm [9]. *P. aeruginosa* uses a dual resistance mechanism: reduced penetration and active drug efflux [10,11]. Moreover, bacteria embedded in the biofilm develop tolerance against high antibiotic concentrations [12]. The *ndvB* gene encodes glucosyltransferase that is involved in the synthesis of cyclic glucagon situated at the periplasm. *P. aeruginosa* cyclic glucans interact with antibiotics and sequester them in the periplasm [13]. Moreover, *P. aeruginosa* also contains adenosine triphosphate-binding cassette (ABC) transport system [14]. ABC transport system includes PA1874, PA1875, PA1876, and PA1877 (PA1874-77) that remove antibiotics from the cells within the biofilm [9].

Young biofilms are more susceptible to antibiotics compared to more developed biofilms [15]. Thus, early intervention could be useful regarding the management of intractable biofilm-associated persistent infections. Very few studies evaluated the accuracy of different methods for the detection of biofilm. Molecular methods are more sensitive and specific. However, no study on the genes responsible for biofilm formation and biofilm-associated antimicrobial resistance among clinical isolates of *P. aeruginosa* in Bangladesh. Therefore, this study was conducted to detect biofilm, and genes responsible for biofilm and antibiotic resistance among *P. aeruginosa.*

## Materials and methods

### Sample collection

This cross-sectional study was conducted in the Department of Microbiology of Dhaka Medical College (DMC), Dhaka, Bangladesh, from July 2017 to December 2018. Among 446 samples (infected burn, surgical wounds, and endotracheal aspirate [ETA]), a total of 232 clinical isolates of *Pseudomonas aeruginosa* from the infected burn, surgical wounds, and ETA were included.

### Bacterial isolation

After collecting aseptically, samples were inoculated in blood agar media and MacConkey agar media followed by incubation aerobically at 37°C and 42°C for 48 hours. *P. aeruginosa* was isolated and identified by colony morphology, Gram staining, and biochemical tests following standard procedures [16]. Identification of *P. aeruginosa* was confirmed by polymerase chain reaction (PCR) from the culture with specific primers [17].

### Antimicrobial susceptibility testing

Isolates were tested for antimicrobial susceptibility using Kirby-Bauer modified disk diffusion method and agar dilution method of minimal inhibitory concentration [18]. Antibiotic susceptibility was interpreted following Clinical and Laboratory Standards Institute guideline, 2017 [19].

### Method of detection of biofilm

*Congo red agar (CRA) method:* CRA medium was prepared with brain heart infusion broth 37 g/L, sucrose 50 g/L, agar No. 1 10 g/L, and Congo red indicator eight g/L. Congo red stain and brain heart infusion agar with sucrose were autoclaved separately followed by mixing at 55°C. CRA plates were inoculated with test organisms and incubated at 37°C for 24 hours aerobically. Black colonies with a dry crystalline consistency indicated biofilm production [20]. The experiment was performed in triplicate and repeated three times.

### Tube method

A loopful of test organisms was inoculated in 10 mL of Trypticase Soy Broth with 1% glucose in test tubes. The tubes were incubated at 37°C for 24 h. After incubation, tubes were decanted and washed with phosphate-buffered saline (PBS) (pH 7.3) and dried. Tubes were then stained with crystal violet (0.1%). The excess stain was washed with deionized water and dried in the inverted position. The scoring for the tube method was done according to the results of the control strains. Biofilm formation was considered positive when a visible film lined the wall and the bottom of the tube. The amount of biofilm formed was scored as 1-weak/none, 2-moderate, and 3-high/strong. The experiment was performed in triplicate and repeated three times [21].

### Tissue culture plate method

Cultures were transferred into a fresh medium in a dilution of 1:100. Ninety-six well flat bottom polystyrene treated tissue culture plates (TCP) (SigmaAldrich, Costar, USA) were filled with 200 µL of diluted cultures. Control organisms were also incubated, diluted, and added to the TCP. The sterile broth was taken as a negative control. After incubation at 37°C for 24 hours, the contents of each well were removed by gentle tapping and washed with 0.2 mL PBS four times to remove free-floating bacteria. Biofilm formed by bacteria adherent to the wells was fixed by 2% sodium acetate and stained by crystal violet (0.1%). The excess stain was removed with deionized water and dried. Optical density (OD) of stained adherent biofilm was obtained using a micro-ELISA auto reader (model 680, Biorad, UK) at wavelength 570 nm. The experiment was performed in triplicate and repeated three times [22].

### Calculation of OD values

The average OD values were calculated for all tested strains and negative controls since all tests were performed in triplicate and repeated three times. Second, the cut-off value (ODc) was established. It was defined as three standard deviations (SD) above the mean OD of the negative control: ODc = average OD of negative controls + (3 × SD of negative control) [23]. It was interpreted as, if average OD ≤ ODc, no biofilm was produced, if ODc < OD ≤ 2ODc, weak biofilm was produced, 2ODc < OD ≤ 4ODc, moderate biofilm was produced, if 4ODc < OD, then high biofilm was produced [23].

### Polymerase chain reaction

*Deoxyribonucleic acid (DNA) extraction:* Bacterial pellets were mixed with 300μl distilled water followed by boiling at 100°C for 10 minutes in a block heater (DAIHA Scientific, Seoul, Korea). After cooling on the ice pack, the mixture was centrifuged at 4°C at 13,500g for 10 minutes. The extracted DNA was then kept at -20°C [24].

*Amplification:* After making a 25μl mixture of mastermix, primer, and DNA template, PCR was performed in DNA thermal cycler (Eppendorf AG, Mastercycler gradient, Hamburg, Germany). The amplified DNA was analyzed by 1.5% agarose gel-electrophoresis at 100 volts for 35 minutes, stained with 1% ethidium bromide, and visualized under an ultraviolet transilluminator (Gel Doc, Major science, Taiwan).

Primer for *P. aeruginosa* species identification was PA-SS (F-GGGGGATCTTCGGACCTCA, R-TCCTTAGAGTGCCCACCCG) with 956bp [17]. Primers for biofilm-forming genes were *lasR* (F-AAGTGGAAAATTGGAGTGGAG, R-GTAGTTGCCGACGACGATGAAG) with 130bp, *pqsA* (F-CCCGATACCGCCGTTTATCA, R-AACCCGAGGTGTATTGCAGG) with 448bp, *pelA* (F-CCTTCAGCCATCCGTTCTTCT, R-TCGCGTACGAAGTCGACCTT) with 118bp, *pslA* (F-TGGGTCTTCAAGTTCCGCTC, R-ATGCTGGTCTTGCGGATGAA), with 119bp, *pslD* (F-CTCATGAAACGCACCCTCCT, R-TGCGACCGATGAACGGATAG) with 295bp, *pslH* (F-CAGATGCTGGTCTGGGAGTG, R-GGAACGAAGCCTTGCCATTC), with 719bp [[25], [26], [27]]. Primers for biofilm-specific antimicrobial resistance genes were *ndvB* (F-GAGGTGGCAAAATGGGCAAG, R-CATGCAGGCAAGAATCGACG) with 781bp, PA1874 (F-GGCCATTACACGATCCACTC, R-GGCTGTATGCAGACCGAAC), with 183bp, PA1876 (F-GATTGTCGGAGGGTCAGAAA, R-CGACACCAGTTGCAGAAATG), with 200bp, PA1877 (F-GCCACAAAATCGAGGAAAAG, R-CGCCAATCGTTGTGATGTAG), with 186bp [28,29]. PA1874, PA1876 and PA1877 are orthologous of BPSL1661, BPSL1664 and BPSL1665 respectively [29]. Gene sequences were used accordingly.

### Data analysis

Data analysis was done by using ‘Microsoft Office Excel 2010’ program. The test of significance was calculated using chi-square test, and *P* value <0.05 was taken as minimal level of significance.

## Results

A total of 446 samples were collected, among which 232 (52.02%) yielded growth of *P. aeruginosa*. Detection of biofilm production in different methods by these *P. aeruginosa* from different samples is shown in Table 1.

**Table 1:** Detection of biofilm production in different methods by *P. aeruginosa* from different samples (N = 232).

Samples	Methods of biofilm-formation		
	TM, n (%)	TCP method, n (%)	CRA method, n (%)
Burn wound (N=126)	33 (26.19)	21 (16.67)	12 (9.52)
Surgical/traumatic wound (N= 95)	0	0	0
ETA (N=11)	3 (27.27)	3 (27.27)	0 (0.00)
Total (N=232)	36 (15.51)	24 (10.34)	12 (5.17)

Source: Original.

CRA, Congo red agar; ETA, endotracheal aspirate; n, number of bacteria forming biofilm in respective method; N, total number of samples; TCP, tissue culture plate; TM, tube method.

Considering TCP as the gold standard method, comparisons of diagnostic parameters (sensitivity, specificity, positive and negative predictive value, and accuracy) between TM and CRA methods are demonstrated in Table 2.

**Table 2:** Diagnostic parameters of tube method and Congo red agar method for biofilm detection.

Screening method	Sensitivity (%)	Specificity (%)	Positive predictive value (%)	Negative protective value (%)	Accuracy (%)
TM	100%	94%	66.67%	100%	94%
CRA	50%	100%	100%	94.55%	94.83%

Source: Original.

CRA, Congo red agar; TM, tube method.

In Table 3, the distribution of biofilm-forming genes among 24 biofilm-producing isolates in the TCP method is shown. *pqsA* was the most common gene isolated from biofilm-forming *P. aeruginosa* isolated from both burn and ETA samples, followed by *pslA* and *pelA*.

**Table 3:** Proportion of biofilm-forming genes among biofilm-forming *P. aeruginosa* detected by tissue culture plate method.

Biofilm-forming genes	Burn wounds (N = 21) n (%)	ETA (N = 3) n (%)	Total (N = 24) n (%)
*pqsA*	17 (80.95)	2 (66.67)	19 (79.17)
*pslA*	16 (76.19)	1 (33.33)	17 (70.83)
*pslD*	11 (52.38)	0 (0)	11 (45.83)
*pslH*	9 (42.86)	0 (0)	9 (37.5)
*pelA*	11 (52.38)	1 (33.33)	12 (50)
*lasR*	8 (33.33)	1 (33.33)	9 (37.5)

Source: Original.

ETA, endotracheal aspirate; N, total number of samples; n, number of *P. aeruginosa* having respective biofilm-producing genes.

Among 24 biofilm-producing isolates in TCP method, four (16.67%) were positive for *ndvB*, two (8.33%) for PA1874, and two (8.33%) for PA1877. All were isolated from burn wound samples. No PA1876 was detected.

Antibiotic resistance pattern of all the isolated *P. aeruginosa* (N = 232)*,* biofilm-forming (N = 24), and non-biofilm-forming (N = 208) is demonstrated in Table 4. Though a higher percentage of resistance was observed to most antibiotics among biofilm-forming stains, resistance to cefotaxime was statistically significant in non-biofilm-forming strains, whereas resistance to colistin was statistically significant among biofilm-forming *P. aeruginosa* (Table 4).

**Table 4:** Comparison of antibiotic resistance patterns between biofilm positive (N = 24) and biofilm negative (N = 208) *P. aeruginosa*.

Antimicrobial agents	Resistance pattern in biofilm-positive strains n (%)	Resistance pattern in biofilm-negative strains n (%)	*P* value
Imipenem	7 (29.17)	65 (30.28)	>0.05
Piperacillin/Tazobactam	13 (54.17)	92 (44.23)	>0.05
Amikacin	20 (83.33)	172 (82.69)	>0.05
Gentamicin	22 (91.67)	185 (88.94)	>0.05
Netilmicin	16 (66.67)	149 (71.64)	>0.05
Aztreonam	18 (75)	155 (74.52)	>0.05
Ciprofloxacin	22 (91.67)	187 (89.90)	>0.05
Cefotaxime	21 (87.5)	202 (97.11)	<0.05^b^
Ceftazidime	17 (70.83)	142 (68.27)	>0.05
Colistin	4 (16.67)	12 (5.77)	<0.05^a^

Source: Original.

a Resistance to Colistin is statistically significant in biofilm positive *P. aeruginosa*.

b Resistance to Cefotaxime is statistically significant in biofilm negative *P. aeruginosa*.

## Discussion

Biofilm-related infections are subject to develop recurrent and chronic wound infections, and device-related infections. Besides, biofilm-related bacterial infection increases mortality rate in burn patients [30].

Detection of biofilm is crucial for the prevention of treatment failure, as it is a threat to hospital-acquired infections [31]. Although the molecular method is more sensitive and specific, phenotypic methods are comparatively less expensive and easier to perform. Sultan and Nabiel reported higher sensitivity and specificity in the tube method, which contradicted our study [32]. In this study, considering TCP method as the gold standard, the tube method showed cent per cent negative predictive value, and the CRA method displayed 100% positive predictive value. Sultana found a higher percentage of biofilm production in Dhaka Medical College and Hospital (61.54% in TCP, 50% in TM, 11.84% in CRA method) [33]. There is no clear explanation for such variations in these studies, but it can be due to batch-to-batch variation of media and subjective judgment of interpretation as TM and CRA are qualitative tests [32].

Biofilms adhere to the human cell surface as a community of microorganisms. These organisms are embedded in the matrix of EPS, self-produced by these adherent cells. pel and psl operons biosynthesize the EPS and help in the interactions between cells during biofilm formation [34]. In current study, biofilm-forming gene *pqsA* (79.17%) was the most prevalent in *P. aeruginosa* isolated from both burn wound and ETA samples. The presence of *pslA* (70.83%), *pslD* (45.83%), *pslH* (37.5%) in biofilm-forming *P. aeruginosa* isolated from burn wound samples was statistically significant. Rajabi et al. reported significant association of psl and pel genes with biofilm formation [35]. Furthermore, *LasR* is a major regulator of QS system in *P. aeruginosa.* However, *LasR* gene and *pqsA* gene do not necessarily co-exists [36]. In this study, LasR gene was less prevalent among biofilm-forming strains. Though biofilm formation is a multistage process that is attributed to many factors, these genes may act as markers for screening biofilm-forming bacteria.

The resistance in biofilms is multifactorial, including the diffusion barrier and efflux transporter in *P. aeruginosa* [37]. Zhang et al. described mutations within the *ndvB* gene of *P. aeruginosa*, which encodes a glucosyltransferase, resulting in increased sensitivity of *P. aeruginosa* biofilms to several antibiotics [38]. In this study, 16.67% of biofilm-forming *P. aeruginosa* were positive for biofilm-specific antimicrobial resistance gene *ndvB*, followed by 8.33% for PA1874 and PA1877, which are not substantial. Besides, resistance to cefotaxime was statistically significant in non-biofilm-forming *P. aeruginosa* compared to biofilm-forming strains. In contrast, colistin resistance among biofilm-forming *P. aeruginosa* was statistically significant (p< 0.05) compared to non-biofilm-forming isolates. So, it was inconclusive whether there was association between biofilm formation and antibiotic resistance in *P. aeruginosa* in this study. Because resistance patterns of other antibiotics among biofilm-forming and non-biofilm-forming *P. aeruginosa* isolated in this study were almost similar. A lower number of biofilm-forming isolates could be responsible for this matter.

## Conclusions

Biofilm-forming *Pseudomonas aeruginosa* was detected from infected burn wounds and ETA samples by TCP method. Detection of biofilm-forming genes can assist in screening biofilm-producing bacteria. Both biofilm-forming and non-biofilm-forming *P. aeruginosa* were resistant to common antibiotics. *ndvB,* PA1874, PA1876, and PA1877 may have a role in antibiotic resistance. However, biofilm-specific antibiotic resistance encompasses multiple mechanisms. Due to time and resource constraints, other mechanisms of antibiotic resistance related to biofilm could not be evaluated. Furthermore, the biofilm detection methods of bacteria were conducted *in vitro*, and it is still unknown what will occur if biofilm is present *in vivo*. Nevertheless, biofilm detection in routine laboratory tests could minimize nosocomial infections. It will help the clinicians to select the proper antibiotics for appropriate patients.

## Declaration of competing interest

The authors have no competing interests to declare.
